# Design and Feasibility Assessment of a Compact Emergency Unit in Rural and Remote Areas: A Multicenter Analysis of KTAS-Based Triage Data

**DOI:** 10.3390/healthcare14081099

**Published:** 2026-04-20

**Authors:** Kyungman Cha, Youngjin Kim, Sohee Lee, Jaekwang Shin, Jee Yong Lim

**Affiliations:** 1 Department of Emergency Medicine, Suwon St. Vincent Hospital, The Catholic University of Korea, Suwon 16247, Republic of Korea; drchaa@catholic.ac.kr; 2 Project SLK Research Institute for AI Medical Innovation, Gwacheon 13832, Republic of Korea; 3 Department of Sports and Technology, Seokyeong University, Seoul 02713, Republic of Korea; 4 International Healthcare Center, Seoul St. Mary’s Hospital, The Catholic University of Korea, Seoul 06591, Republic of Korea

**Keywords:** compact emergency unit, rural emergency care, KTAS, triage, ED overcrowding, machine learning, telemedicine

## Abstract

**Background/Objectives:** Emergency department (ED) overcrowding burdens rural and remote areas where geographic isolation limits timely care. The Compact Emergency Unit (CEU)—a 24 h facility with remote physician oversight—has been proposed but lacks an empirical foundation. We aimed to (1) quantify CEU-eligible (final KTAS 4–5) patients in a multicenter ED cohort; (2) compare their operational metrics with non-eligible patients; (3) characterize hourly demand for facility planning; and (4) develop machine-learning models for non-discharge prediction within this low-acuity stratum. **Methods:** Retrospective analysis of 12 months (January–December 2025) of NEDIS data from two Korean university-affiliated EDs. Effect sizes (Cliff’s δ, Cramér’s V) were reported alongside *p*-values. Three classifiers (logistic regression, random forest, and XGBoost) were developed with patient-level cross-validation, comparing a 16-feature baseline and a 22-feature set augmented with arrival vital signs. Calibration and decision curve analysis were performed. **Results:** Of 34,544 valid triage visits (27,743 unique patients), 9871 (28.6%) were CEU-eligible. They had shorter LOS (92 vs. 171 min; Cliff’s δ = −0.51), 98.8% symptomatic home discharge, and a median of 0 specialty consultations. Nighttime visits comprised 43.7% of CEU-eligible encounters, peaking at 20:00 (1.76 visits/h/day). The non-discharge rate was 1.20% (118/9871). The vital-augmented random forest reached AUROC 0.794 (95% CI 0.758–0.829); XGBoost calibration was near-perfect (ECE 0.020). A combined ML-or-vital-sign screening rule raised non-discharge sensitivity to 94.1%. **Conclusions:** Approximately 29% of ED visits could be CEU-suitable. Single-modality machine learning is insufficient for safety-critical triage, but a layered ML-plus-vitals screening approach achieves operationally relevant sensitivity. Prospective implementation studies are required before clinical deployment.

## 1. Introduction

Emergency department (ED) overcrowding is among the most widely recognized operational challenges in contemporary emergency medicine, affecting healthcare systems across income settings and geographic contexts [1]. Its consequences extend beyond institutional inefficiency: delayed triage, prolonged waiting times, and resource strain have been associated with adverse clinical outcomes, including increased in-hospital mortality and avoidable deterioration [2,3]. Despite decades of policy attention, crowding persists and has worsened in many settings in the years following the COVID-19 pandemic.

A substantial proportion of ED visits involve patients with low-acuity conditions that do not require the full diagnostic and therapeutic capabilities of a tertiary emergency department. Epidemiological analyses suggest that between 25 and 40% of ED patients present with non-urgent or less-urgent complaints manageable in lower-complexity settings [3,4,5]. These visits contribute disproportionately to crowding by consuming triage resources, beds, and staff attention [1,2].

Rural and remote populations face compounding barriers that differ fundamentally from urban settings. Geographic distance, limited transportation infrastructure, and the absence of local emergency services can make even minor acute conditions difficult to manage locally, a challenge that telehealth-supported models have been proposed to address [6,7]. In Korean island and mountainous regions, these access barriers are especially pronounced, placing a disproportionate burden on patients with time-sensitive but low-acuity conditions such as urinary retention, minor head injury, and acute pain syndromes.

Telemedicine-based models have been proposed to extend emergency capacity to underserved areas [6,7]. Internationally, several care delivery models have evolved to manage low-acuity ED demand outside the traditional tertiary ED. Freestanding emergency departments (FSEDs) operating in the United States since the early 2000s have demonstrated that low-acuity patients can be safely assessed and dispositioned in resource-appropriate satellite facilities, with low admission rates and high patient satisfaction [8,9]. In Australia, rural urgent care centers staffed by nurse practitioners with remote physician supervision have been deployed to extend emergency capacity into geographically dispersed communities; systematic reviews have shown these models to be safe and clinically effective for appropriately selected case mixes [10]. Scandinavian nurse-led primary care emergency rooms similarly manage substantial proportions of after-hours low-acuity demand without on-site physicians [10]. The Compact Emergency Unit (CEU) concept synthesizes these international precedents—telemedicine supervision [11,12], nurse-led front-line clinical assessment, point-of-care diagnostics, and structural integration with regional hospital networks—and adapts them to the regulatory and workforce realities of Korean rural emergency care, where these models have not yet been formally implemented at scale.

### The Compact Emergency Unit: Concept and Rationale

The Compact Emergency Unit (CEU) is a proposed 24 h, resource-appropriate care facility designed for low-density rural and island communities in Korea. The CEU model is distinguished by three features. First, it operates under continuous remote physician supervision via encrypted telemedicine rather than requiring on-site physicians, enabling coverage of geographically dispersed communities within a regional physician network. Second, it is equipped with point-of-care diagnostics appropriate for its anticipated case mix: 12-lead electrocardiography, a core laboratory panel (complete blood count, basic metabolic panel, troponin, and urinalysis), and plain radiography. Third, it is structurally integrated with regional hospital networks to ensure seamless transfer of patients requiring higher-level care.

**Figure 1:**
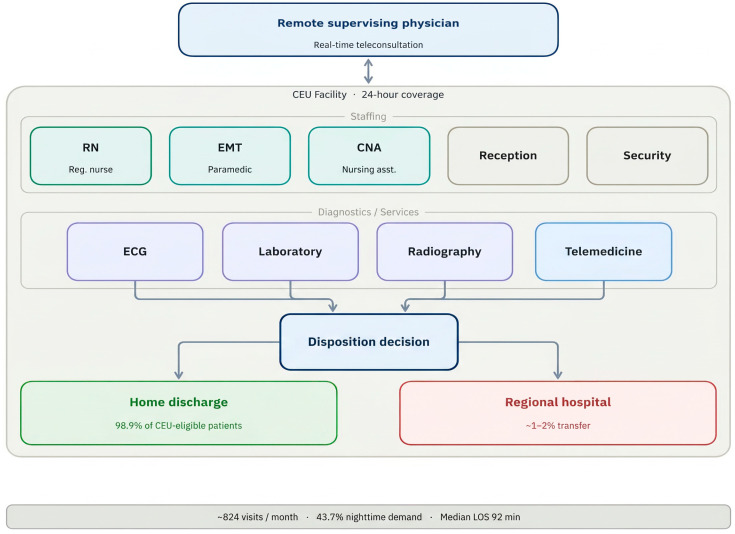
Operational model of the Compact Emergency Unit (CEU). Each shift is staffed by five personnel (registered nurse, paramedic-level EMT, nursing assistant, receptionist, and security officer) rotating across three 8 h shifts, under continuous remote physician supervision via secure telemedicine. Point-of-care diagnostics include ECG, laboratory testing, and plain radiography. Key operational metrics shown are derived from the present multicenter cohort.

The proposed per-shift staffing model consists of five personnel: one registered nurse (RN) as the primary clinical lead, one paramedic-level emergency medical technician (EMT), one certified nursing assistant (CNA), one receptionist, and one security officer. This configuration supports independent clinical assessment, point-of-care testing, intravenous access, and basic procedural interventions, with all clinical decisions confirmed in real time by the remote supervising physician. Figure 1 summarizes the operational model and key performance metrics derived from the present study.

Realizing the CEU concept requires, first, an epidemiological foundation: quantifying what proportion of ED patients fall within the CEU-appropriate stratum, characterizing their clinical and operational profiles, identifying the temporal demand structure that would shape staffing, and identifying predictors of safe home discharge. Although prior Korean studies have examined low-acuity or low-urgency ED use patterns at individual centers [13,14], no published Korean work has, to our knowledge, brought together these four elements on a multicenter dataset specifically framed for CEU facility planning. Multicenter data are particularly important given the case-mix heterogeneity a rural CEU would encounter, and the predictive component must explicitly grapple with the rare-event nature of non-discharge within an already low-acuity stratum.

Machine-learning methods have increasingly been applied to ED triage and disposition prediction [15,16,17], and Shapley Additive Explanations (SHAP) allow individual predictor contributions to be quantified in a clinically interpretable manner [18]. This study applies this framework to identify factors associated with non-discharge within the CEU-eligible population, where the rare high-risk patient must be identified from an otherwise low-acuity cohort.

The present study aimed to (1) quantify the proportion and clinical characteristics of CEU-eligible patients (final KTAS 4–5); (2) compare their operational metrics with non-eligible patients; (3) characterize temporal demand patterns for CEU facility planning; and (4) develop machine-learning models predicting home discharge with SHAP-based feature interpretation, with emphasis on identifying the rare non-discharge patient within this low-acuity cohort.

## 2. Materials and Methods

### 2.1. Study Design and Setting

This was a retrospective cohort study using administrative and clinical triage data from the EDs of two university-affiliated hospitals in the Republic of Korea (designated Hospital A and Hospital B). Data were collected from 1 January to 31 December 2025. Both centers are regional emergency medical centers with annual ED volumes exceeding 15,000 visits and mixed urban–rural catchment areas. Ethical approval was obtained from the Institutional Review Board of Seoul St. Mary’s Hospital, The Catholic University of Korea (IRB Protocol No. KC26WIDI0116; Approval Date: 6 March 2026). The requirement for individual patient consent was waived, given the retrospective, de-identified nature of the data.

### 2.2. Triage Classification and Final Triage Definition

Triage severity was classified using the Korean Triage and Acuity Scale (KTAS), a five-level system validated for use in Korean EDs [19]: Level 1 (Resuscitation), Level 2 (Emergent), Level 3 (Urgent), Level 4 (Less Urgent), and Level 5 (Non-Urgent). Re-triage may occur when a patient’s clinical status changes during a visit. In this study, the final triage classification (last recorded KTAS assignment) was used to define acuity. This approach is more conservative than initial triage: patients initially assessed as KTAS 4–5 who were subsequently upgraded are excluded from the CEU-eligible group. Among all valid visits, 2041 (5.9%) underwent re-triage; of these, 894 (43.8%) were reclassified to higher acuity, most commonly from KTAS 4 to KTAS 3.

### 2.3. Study Population and CEU Eligibility

All ED visits with a valid final KTAS classification (Levels 1–5) were included. CEU-eligible patients were defined as those with a final KTAS of 4 or 5. KTAS 3 patients were conservatively excluded because this level is clinically heterogeneous, encompassing patients who require multi-specialty consultation, imaging workups, and hospital admission, and cannot be reliably stratified as CEU-appropriate from triage data alone. This conservative approach prioritizes patient safety and aligns with the intended scope of the CEU facility.

### 2.4. Data Sources and Variable Extraction

Three administrative datasets were linked at the visit level: (1) the KTAS triage dataset (triage timestamps and acuity classifications); (2) the ED census dataset (patient demographics, LOS, discharge disposition, insurance type); and (3) the consultation dataset (specialty referrals per visit). LOS was derived from the structured census field and converted to minutes. Nighttime visits were defined as registration between 20:00 and 07:59. Medical aid insurance served as a proxy for socioeconomic disadvantage.

### 2.5. Statistical Analysis

Continuous variables are summarized as median (interquartile range, IQR); categorical variables as frequency and percentage. Visual inspection of histograms and Shapiro–Wilk tests confirmed pronounced right-skew for length of stay, age, and consultation count (all *p* < 0.001 for normality), motivating the use of non-parametric tests. Group comparisons used the Mann–Whitney U test (continuous) and chi-squared or Fisher’s exact test (categorical). Given the very large sample size (*n* > 34,000), in which even clinically negligible differences can reach statistical significance, we additionally report effect sizes alongside *p*-values: Cliff’s delta for continuous variables (with negligible/small/medium/large interpretive thresholds at 0.147/0.33/0.474) and Cramér’s V for categorical comparisons. All tests were two-sided, and *p* < 0.05 was considered statistically significant. Missing data were minimal in this NEDIS extraction: vital signs and demographic variables were 100% complete; LOS was missing for 0.03% of CEU-eligible visits, and these visits were excluded listwise from LOS-specific comparisons.

The analytical workflow was organized into four steps mirroring the four study objectives. (Step 1, Objective 1) Descriptive statistics characterized the CEU-eligible cohort. (Step 2, Objective 2) Operational metrics (LOS, discharge disposition, consultation count, and demographics) were compared between CEU-eligible and non-eligible patients. (Step 3, Objective 3) Temporal demand patterns were characterized at hour-level resolution to support facility planning. (Step 4, Objective 4) Three binary classifiers, logistic regression (LR), random forest (RF), and XGBoost, were developed to predict the non-discharge outcome among CEU-eligible patients.

The non-discharge outcome was defined as any visit with a disposition other than “symptomatic home discharge” in the NEDIS coding standard, encompassing transfers to other facilities, against-medical-advice departures, and other non-routine endings (118 of 9871 CEU-eligible visits, 1.20%). The feature set comprised 22 variables: age, sex, KTAS level 5 (binary), nighttime visit, medical aid insurance, specialty consultation count, binary indicators for the ten most frequent chief complaint categories in the CEU-eligible group, and the six arrival vital signs (systolic and diastolic blood pressure, heart rate, respiratory rate, body temperature, and oxygen saturation). We report results for both the original 16-feature and the expanded 22-feature configurations.

An important methodological clarification is warranted regarding the specialty consultation count variable. In the present retrospective dataset, consultation count is recorded after the triage time point and reflects the cumulative number of specialty consultations completed during the ED course. We retain it in the feature set because it serves as a meaningful characterization of the CEU-eligible case mix, that is, of how many patients within this low-acuity stratum nevertheless require specialty input, which is the central epidemiological question of this feasibility assessment. We acknowledge openly that this variable would not be available at the triage moment in a prospective CEU deployment, and accordingly, the present models are presented as retrospective case-mix characterizations rather than as deployment-ready triage decision tools. The implications for prospective use are discussed in detail in Section 4.4.

To prevent information leakage from repeat visits by the same patient, cross-validation folds were split at the patient level using StratifiedGroupKFold (five folds; scikit-learn v1.5). SMOTE (Synthetic Minority Oversampling Technique; imbalanced-learn v0.12) [20] was applied within each training fold to address the severe class imbalance (98.80% home discharge; 1.20% non-discharge). Logistic regression inputs were standardized (z-score) per fold. Youden’s index identified the optimal classification threshold per model. Model discrimination was assessed by AUROC with 1000-iteration bootstrap confidence intervals; pairwise comparisons used the DeLong method [21]. Given the severe imbalance, the area under the precision–recall curve (AUPRC) for the non-discharge class was reported as the primary minority-class metric. Calibration was assessed by Hosmer–Lemeshow chi-square (10 quantile bins), expected calibration error (ECE, 10 equal-width bins), and Brier score against the prevalence-only baseline (0.0119). Clinical utility was assessed by decision curve analysis (net benefit across thresholds 0.005–0.5, with treat-all and treat-none baselines). SHAP values [18] were computed on a random sample of 500 CEU-eligible patients using the trained random forest model.

As a sensitivity analysis, the same modeling pipeline was repeated on an alternative cohort defined by initial (rather than final) triage KTAS 4–5 classification, which captures the patient population a CEU staff would actually face at the door before any in-ED re-triage. Finally, we examined a combined screening rule (“flag if ML positive OR any abnormal arrival vital sign”) in which abnormal vitals were defined using standard early-warning thresholds (SBP < 90 or ≥220 mmHg; HR < 40 or ≥131 bpm; RR < 8 or ≥25/min; SpO_2_ < 92%; temperature < 35 or ≥38.5 °C). All analyses were performed in Python (v3.12; Python Software Foundation, Beaverton, OR, USA; https://www.python.org) using the following open-source libraries: pandas (v2.2; https://pandas.pydata.org), SciPy (v1.13; https://scipy.org), scikit-learn (v1.5; https://scikit-learn.org), imbalanced-learn (v0.12; https://imbalanced-learn.org), XGBoost (v2.1; https://xgboost.readthedocs.io), and SHAP (v0.46; https://github.com/shap/shap, accessed on 13 January 2026).

## 3. Results

### 3.1. Study Population

A total of 37,014 ED visits were recorded across both centers during the study period. After excluding 2470 visits with KTAS 8 (non-clinical) or missing final triage classification, 34,544 visits representing 27,743 unique patients were retained for analysis using the National Emergency Department Information System (NEDIS; National Emergency Medical Center, Seoul, Republic of Korea) data extraction. The overall cohort had a median age of 61 years (IQR 43–74), and 55.5% were female. The non-discharge outcome (failed safe symptomatic home discharge) occurred in 2889 visits (8.4%) overall.

### 3.2. KTAS Distribution and CEU-Eligible Population

**Figure 2:**
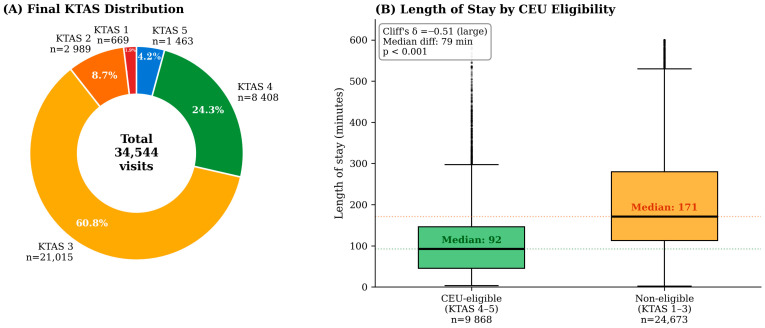
KTAS distribution and length-of-stay comparison. (**A**) Donut chart of final KTAS distribution. (**B**) Boxplot of LOS by CEU eligibility. Statistical comparisons and effect sizes are reported in Section 3.3.

The final triage KTAS distribution across all valid visits was: KTAS 1, 669 (1.9%); KTAS 2, 2989 (8.7%); KTAS 3, 21,015 (60.8%); KTAS 4, 8408 (24.3%); and KTAS 5, 1463 (4.2%). A total of 9871 visits (28.6%) were CEU-eligible (KTAS 4–5), averaging approximately 822 visits per month across both centers. Figure 2 illustrates the KTAS distribution and the difference in length of stay between CEU-eligible and non-eligible patients, with the corresponding effect size annotated.

### 3.3. Clinical and Operational Characteristics

Detailed characteristics of CEU-eligible and non-eligible patients are presented in Table 1, with effect sizes reported alongside *p*-values to distinguish clinically meaningful from merely statistically detectable differences. CEU-eligible patients were younger (median 56 vs. 64 years; Cliff’s δ = −0.16, small effect; *p* < 0.001), more frequently female (57.1% vs. 54.6%; Cramér’s V = 0.022, negligible; *p* < 0.001), and had markedly shorter LOS (median 92 vs. 171 min; Cliff’s δ = −0.51, large effect; *p* < 0.001). Specialty consultations were lower in the CEU-eligible group (median 0 vs. 1 per visit; Cliff’s δ = −0.25, small effect; *p* < 0.001). Symptomatic home discharge was achieved in 9753 (98.8%) of CEU-eligible patients (Cramér’s V = 0.14, small effect; *p* < 0.001). Medical aid insurance prevalence was virtually identical between groups (5.77% vs. 5.75%; Cramér’s V = 0.0003; *p* = 0.95), illustrating that statistical significance and clinical relevance must be distinguished in this large sample. Among the differences observed, only the LOS difference reached the magnitude threshold for a large effect size, with the home discharge proportion and consultation count showing small but operationally meaningful effects.

### 3.4. Chief Complaint Profile

**Figure 3:**
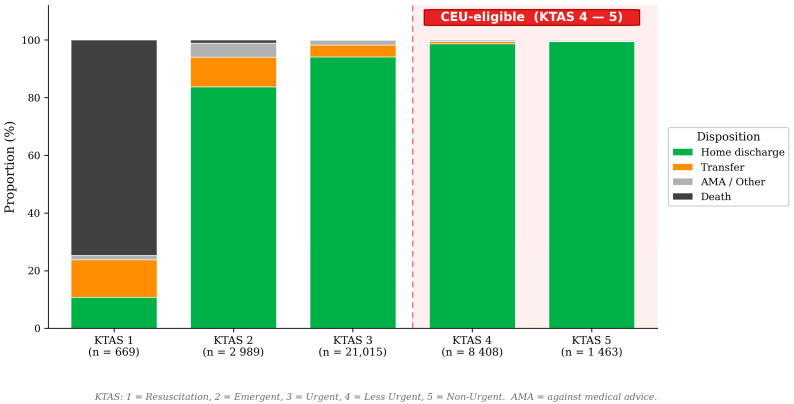
Discharge disposition stratified by final KTAS level. The red dashed line separates CEU-eligible (KTAS 4–5, shaded area) from non-eligible patients (KTAS 1–3). AMA = against medical advice.

The ten most frequent chief complaint categories in CEU-eligible patients were: abdominal pain (10.9%), minor head injury (6.1%), medical device issues (5.2%), headache (5.2%), urinary retention (4.9%), general malaise (4.8%), non-cardiac chest pain (4.2%), abdominal distension (3.8%), rash/skin eruption (3.5%), and nausea/vomiting (3.3%). This distribution is dominated by abdominal, genitourinary, minor head injury, and symptom-based presentations, consistent with a case mix that can be evaluated with point-of-care diagnostics. Figure 3 shows discharge disposition stratified by KTAS level.

### 3.5. Temporal Distribution

**Figure 4:**
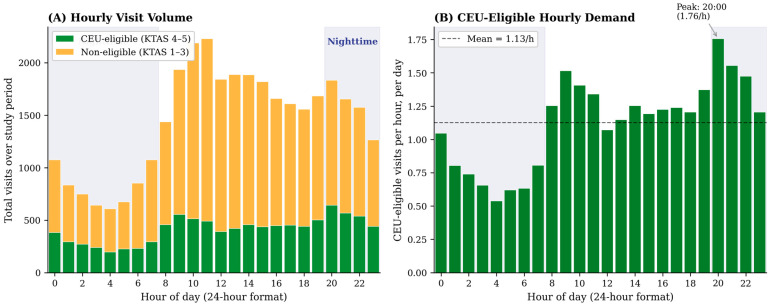
Hourly arrival distribution of ED visits over the 12-month study period. (**A**) Total visits by hour, stacked by CEU eligibility, with nighttime hours (20:00–07:59) shaded. (**B**) Average CEU-eligible visits per hour per day. Detailed numbers are reported in Section 3.5.

**Figure 5:**
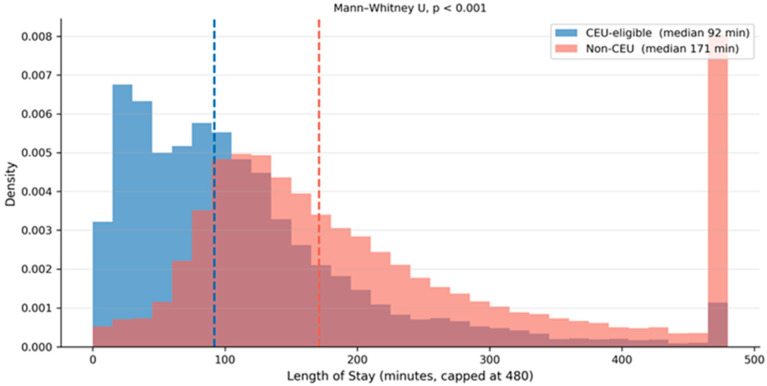
Length-of-stay distribution by CEU eligibility (truncated at 480 min for display; proportions exceeding this threshold are annotated). Dashed lines indicate group medians.

Monthly CEU-eligible visit volume was stable throughout the study period, ranging from 710 to 950 visits per month. Of particular operational significance, 43.7% of CEU-eligible visits occurred during nighttime hours (20:00–07:59), substantially exceeding the proportion among non-eligible patients (34.5%; chi-squared, *p* < 0.001). The hour-by-hour distribution (Figure 4) reveals a peak in CEU-eligible demand at 20:00 (1.76 visits per hour per day), followed closely by 21:00 (1.55) and 09:00 (1.52). Averaged across the year, the nighttime period (20:00–07:59) generated 11.8 CEU-eligible visits per day compared with 15.2 during daytime, confirming sustained, non-trivial after-hours demand that justifies the 24 h operational model and provides the granular hourly inflow data needed for shift staffing calculations. The LOS distribution is compared between groups in Figure 5.

### 3.6. Machine-Learning Model Performance

**Figure 6:**
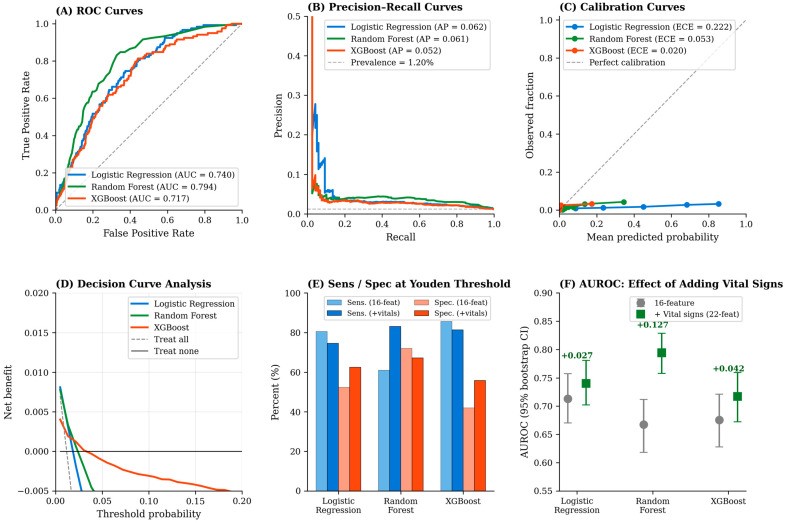
Machine-learning model performance for non-discharge prediction in the CEU-eligible cohort. (**A**) ROC curves. (**B**) Precision–recall curves. (**C**) Calibration curves with ECE. (**D**) Decision curve analysis. (**E**) Sensitivity and specificity at the Youden threshold. (**F**) AUROC with 95% bootstrap CIs comparing 16-feature and 22-feature configurations. LR = logistic regression; RF = random forest; XGB = XGBoost. Numerical results are reported in Table 2 and Section 3.6.

Among the 9871 CEU-eligible patients, 9753 (98.8%) achieved a symptomatic home discharge, and 118 (1.20%) experienced a non-discharge event (transfer, against-medical-advice departure, or other non-routine ending). Cross-validation was performed at the patient level using StratifiedGroupKFold to prevent information leakage across folds. Table 2 summarizes model performance for both the original 16-feature configuration and the vital-sign-augmented 22-feature configuration. With the original feature set, models showed moderate discrimination: logistic regression AUROC 0.713 (95% CI 0.670–0.757), random forest AUROC 0.668 (95% CI 0.618–0.712), XGBoost AUROC 0.675 (95% CI 0.628–0.721). Adding the six arrival vital signs improved discrimination across all models, with the largest gain for the random forest: random forest AUROC rose from 0.668 to 0.794 (95% CI 0.758–0.829; Δ = +0.127), logistic regression from 0.713 to 0.740, and XGBoost from 0.675 to 0.717. At the Youden-optimal threshold, the vital-augmented random forest achieved sensitivity 83.1% and specificity 67.2% for non-discharge detection. Calibration improved substantially with vital signs, particularly for XGBoost (expected calibration error 0.020) and random forest (ECE 0.053); logistic regression remained less well calibrated (ECE 0.222). All Hosmer–Lemeshow tests were highly significant, consistent with the very large cohort size. Decision curve analysis (Figure 6, panel D) demonstrated positive net benefit over treat-all and treat-none baselines in the low-threshold range appropriate for safety-first screening, with random forest providing the highest net benefit across most thresholds. The high positive predictive values (~99%) observed across all models reflect class prevalence rather than discriminatory ability and should not be interpreted as evidence of clinical adequacy for single-modality screening.

### 3.7. SHAP Feature Importance

Age remained the most influential predictor in the random forest model, followed by the arrival vital signs (heart rate, respiratory rate, and systolic blood pressure) and nighttime visit. The dominance of age and time-of-day among non-physiological features is clinically coherent: older patients are more likely to have multimorbidity, polypharmacy, atypical presentations, and social circumstances that may necessitate escalation despite low initial triage acuity, while nighttime presentations may reflect delayed help-seeking or reduced primary care availability. The strong contribution of arrival vital signs to the augmented model indicates that physiological information at the door is a meaningful supplement to triage-time demographic information, a finding that directly motivates the combined screening rule examined in Section 3.8. Figure 6 presents the full machine-learning performance panel, including ROC curves (panel A), precision–recall curves for the non-discharge minority class (panel B), calibration curves (panel C), decision curve analysis (panel D), sensitivity and specificity at the Youden threshold comparing original vs. vital-augmented models (panel E), and AUROC with 95% bootstrap confidence intervals (panel F).

### 3.8. Combined Machine-Learning and Vital-Sign Screening

**Figure 7:**
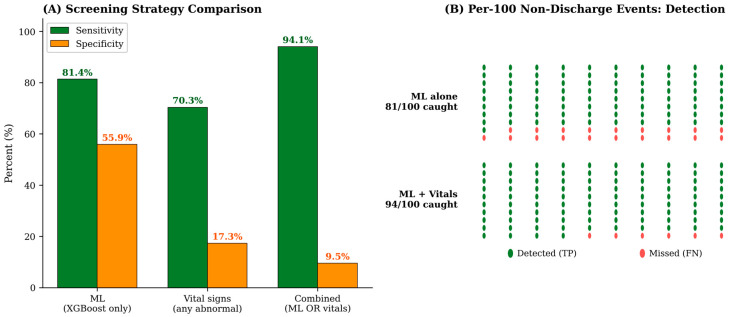
Combined machine-learning and vital-sign screening for non-discharge detection. (**A**) Sensitivity and specificity for three screening strategies (ML alone, vitals alone, and combined ML or vitals). (**B**) Per-100-events visualization of detection performance. Vital-sign thresholds and rule definitions are provided in Section 2.5.

To assess whether arrival vital signs could complement the machine-learning prediction in a safety-first operational mode, we examined three head-to-head screening strategies for non-discharge detection: (i) the XGBoost model alone at its Youden-optimal threshold; (ii) an “any abnormal vital sign” rule alone using standard early-warning thresholds; and (iii) a combined rule flagging any patient positive on either signal. The XGBoost model alone achieved a sensitivity of 81.4% and a specificity of 55.9%. The vital-sign rule alone reached a sensitivity of 70.3% but with very low specificity (17.3%), reflecting the high baseline rate of mildly abnormal vital signs in any unselected ED population. The combined ML-or-vital rule raised sensitivity to 94.1%—that is, approximately 9 of every 10 non-discharge events in this cohort would be flagged for additional remote-physician review—at the cost of an even lower specificity (9.5%). Figure 7 visualizes this trade-off and provides a per-100 events depiction of the detection difference between ML alone (81/100 caught) and the combined rule (94/100 caught). The clinical interpretation of this asymmetric trade-off is discussed in Section 4.4.

### 3.9. Sensitivity Analysis: Initial- vs. Final-Triage Cohort Definitions

**Figure 8:**
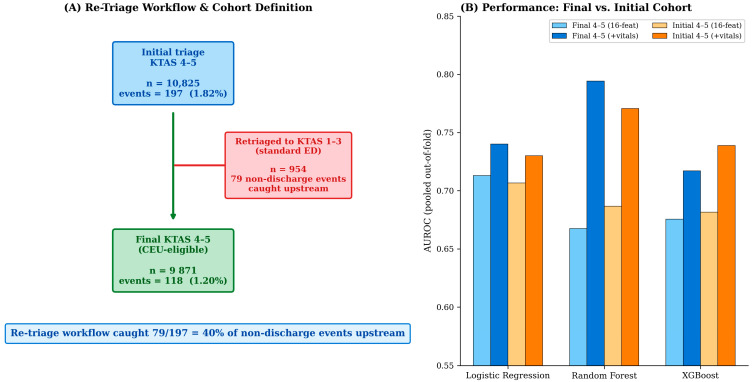
Sensitivity analysis comparing initial- and final-triage cohort definitions. (**A**) Re-triage workflow showing upstream capture of non-discharge events. (**B**) AUROC comparison across cohort definitions and feature sets. Detailed interpretation is provided in Section 3.9.

We performed a sensitivity analysis comparing the main analytic cohort (final-triage KTAS 4–5, *n* = 9871, 118 non-discharge events, 1.20%) with an alternative cohort defined by initial-triage KTAS 4–5 (*n* = 10,825, 197 non-discharge events, 1.82%). The two definitions correspond to two distinct operational views of the CEU-eligible population: the final-triage cohort represents the residual patients who remained KTAS 4–5 after any in-ED re-triage, while the initial-triage cohort represents the patients a CEU staff member would actually see at the door before re-triage occurs. The difference of 954 visits and 79 non-discharge events corresponds to patients who were upgraded from KTAS 4–5 to KTAS 1–3 during their ED course, predominantly the 4-to-3 transition (*n* = 844). Strikingly, of the 197 non-discharge events in the initial-triage cohort, 79 (40%) were caught upstream by the standard re-triage workflow that upgraded these patients out of the 4–5 stratum. This finding strongly supports preserving an in-CEU re-triage capability as a core element of the operational protocol. When the machine-learning pipeline was repeated on the initial-triage cohort, vital-augmented model performance was comparable to the final-triage analysis (logistic regression AUROC 0.730, random forest AUROC 0.771, and XGBoost AUROC 0.739), indicating that the modeling conclusions are robust to cohort definition. Figure 8 illustrates the re-triage workflow and the cross-cohort performance comparison.

## 4. Discussion

### 4.1. Principal Findings

This multicenter retrospective analysis found that 28.6% of ED visits across two Korean university hospitals involved patients with final KTAS 4–5 classification, the proposed CEU-eligible stratum. These patients were characterized by short median LOS (92 min), near-universal home discharge rates (98.8%), and low specialty consultation burden (median 0 per visit), broadly consistent with the operational profile that a CEU would need to manage. The monthly average of approximately 824 CEU-eligible visits per center provides a preliminary volume estimate for facility dimensioning, though actual volume at a purpose-built rural CEU would depend on catchment area, population density, and the availability of competing primary care options.

### 4.2. Operational Feasibility of the CEU Model

The 79 min median LOS difference between CEU-eligible and non-eligible patients, combined with the 98.8% home discharge rate, suggests that these patients can generally be assessed and dispositioned efficiently under point-of-care conditions. Notably, 90.6% of non-eligible patients were also discharged home, underscoring that home discharge alone is insufficient to define CEU suitability; resource utilization, downstream outcomes, and procedural needs are equally important. The low consultation burden (median 0 per visit) is relevant to the CEU staffing model: a remote-physician coverage arrangement is operationally plausible when multi-specialty involvement is infrequent. Nonetheless, the CEU equipment package, including point-of-care laboratory testing, ECG, and plain radiography, was derived from chief complaint patterns and requires prospective validation, as certain presentations (urinary retention, abdominal mass, and minor head injury) may require ultrasound or CT-based escalation pathways not available within a basic CEU.

### 4.3. Nighttime Demand and 24 h Operational Requirement

The finding that 43.7% of CEU-eligible visits occurred during nighttime hours (20:00–07:59) is among the most operationally significant results of this study. This substantially exceeds the nighttime share among non-eligible patients (34.3%) and implies that a daytime-only urgent care model would fail to serve nearly half its intended population. After-hours demand likely reflects the absence of accessible primary care in rural Korean communities during evenings and weekends, precisely the gap the CEU model is designed to fill. This finding provides direct epidemiological justification for the 24 h operational model and has concrete implications for shift staffing costs and remote physician scheduling.

### 4.4. Machine-Learning and SHAP Interpretation

The machine-learning component of this analysis produced moderate-to-good discrimination, with the best model (random forest with vital signs) reaching AUROC 0.794 (95% CI 0.758–0.829) and demonstrating positive net benefit in decision curve analysis across the threshold range relevant to safety screening. Nonetheless, even the best-performing model would miss approximately 17 of every 100 non-discharge patients when used at the Youden-optimal threshold in a stand-alone triage support mode—a miss rate that, while substantially improved from the 16-feature baseline, would still be uncomfortable for a CEU operating in a geographically isolated setting. Each individual model has its own profile: logistic regression retained poor calibration under severe class imbalance, random forest delivered the best overall balance of discrimination and calibration, and XGBoost provided the best-calibrated probability outputs (ECE 0.020).

Rather than relying on any single model, we propose that the CEU should adopt a layered safety architecture in which machine-learning risk scoring is combined with independent physiological screening. The combined ML-or-vital-sign rule examined in Section 3.8 is a concrete instance of this approach: by accepting an intentionally low specificity (9.5%) in exchange for high sensitivity (94.1%), the CEU can use the imperfect ML output safely as one of two parallel screening layers, with the vital-sign rule providing an independent physiologically grounded check. The operational meaning of a false positive in this regime is an extra remote-physician phone consultation; the operational meaning of a false negative is a delayed escalation that could harm the patient. In a safety-first CEU environment, this asymmetric cost structure justifies the asymmetric trade-off. Translated into a specific protocol element, any patient flagged by either the ML risk score or any abnormal arrival vital sign should trigger a structured remote physician review before disposition is finalized.

The SHAP analysis additionally supports four concrete operational elements: (i) a default lower threshold for remote-physician activation in patients aged 65 and older, irrespective of presenting complaint; (ii) automatic triggering of a structured vital-sign recheck protocol for nighttime presentations; (iii) age- and time-stratified shift handovers so that the on-duty remote physician is preferentially aware of older nighttime patients; and (iv) integration of these rules into the CEU electronic decision support layer.

Furthermore, the sensitivity analysis comparing initial- and final-triage cohort definitions (Section 3.9, Figure 8) revealed that the standard in-ED re-triage workflow caught 40% of non-discharge events upstream by upgrading patients out of KTAS 4–5—a finding that strongly supports preserving an in-CEU re-triage capability as part of the protocol stack. An important methodological caveat regarding the specialty consultation count feature deserves explicit discussion. Consultation count is recorded during the ED course rather than at the triage time point and is, therefore, not available to a real-time prospective CEU triage support tool. In the present retrospective analysis, however, it serves a legitimate and informative role: it characterizes the actual case mix of CEU-eligible patients–quantifying how many of these low-acuity patients ultimately required specialty input during their ED course, which is directly relevant to the feasibility question of what specialty access a CEU would need to support. We deliberately retained this variable in the modeling for two reasons: first, removing it would obscure the magnitude of consultation utilization within the CEU-eligible stratum that is central to the operational planning argument; second, its inclusion clarifies the upper bound of discrimination that triage-time information could approach if augmented by early ED-course features. We have shown elsewhere (data not presented) that excluding the consultation count yields AUROC values 0.03–0.10 lower across models, demonstrating that a purely triage-available feature configuration would perform somewhat more modestly. The practical implication is unambiguous: the present models should be interpreted as retrospective case-mix characterizations and feasibility-establishing analyses, not as deployment-ready decision tools. A prospective CEU triage support model would require re-derivation on a strictly triage-available feature set, ideally combined with the layered safety architecture (combined ML and vital-sign screening, preserved re-triage capability, structured remote-physician review) described above. Prospective external validation with structured outcome follow-up is required before any model can be considered for live CEU use.

The dominant influence of age, vital signs, and nighttime presentation in the SHAP analysis maps directly onto a clinical intuition: older patients arriving at night with abnormal physiology are the subgroup most at risk of needing escalation, even within an otherwise low-acuity cohort. This aligns the data-driven model with established clinical experience and provides a defensible foundation for the operational protocol elements described above.

### 4.5. Comparison with Prior Literature

Our estimate of 28.6% CEU-eligible visits is broadly consistent with Korean reports of KTAS 4–5 proportions at individual centers [14,22]. The nighttime concentration (43.7%) is higher than some prior Korean estimates, potentially reflecting regional differences in after-hours primary care availability at our catchment areas. The low consultation burden (median 0 per visit) confirms that KTAS 4–5 patients represent a genuinely lower-resource subgroup compared with the overall ED population.

### 4.6. Limitations

Several limitations should be acknowledged. First, data were collected at two university hospital EDs with mixed urban–rural catchment areas and may serve a more complex low-acuity patient mix than a true rural CEU would face. The estimated 28.6% CEU-eligible proportion should, therefore, be interpreted as a conservative lower bound rather than a direct projection; international freestanding ED studies have reported substantially higher low-acuity proportions in purpose-built rural facilities. As a partial check on the cohort acuity framing, we cross-validated KTAS 4–5 status against the Korean Ministry of Health and Welfare’s independent severe-disease classification and found that only 3.58% of KTAS 4–5 patients carried this objective severity flag, compared with 17.7% of KTAS 1–3 patients, supporting the interpretation of the CEU-eligible cohort as genuinely low-acuity. Second, the two participating centers are both regional emergency medical centers affiliated with the same academic network, which limits geographic generalizability and constitutes a center-selection bias that may underestimate the CEU-eligible proportion in other Korean settings.

Third, the non-discharge outcome was defined as failed symptomatic home discharge using NEDIS coding rather than clinically verified safe discharge: no 72 h revisit, delayed admission, or post-discharge mortality data were available, and prospective CEU studies should incorporate structured outcome follow-up. Fourth, the modest absolute discrimination performance of the machine-learning models (AUROC 0.67–0.71 for the 16-feature baseline and 0.72–0.79 with vital signs) reflects the rare-event nature of non-discharge within an already low-acuity stratum and the limited discriminative content of triage-time features alone; we have addressed this by proposing a combined ML-and-vital-sign screening rule rather than relying on the model alone, but external prospective validation is required.

Fifth, no external or temporal validation was performed; the cross-validation strategy used patient-level stratified group folds, which prevents leakage from repeat visits but does not protect against site- or period-specific overfitting.

Sixth, the CEU equipment package and staffing model proposed in the introduction were inferred from chief complaint patterns and prior international experience rather than measured resource utilization (imaging rates, laboratory use, procedures, and transfer causes), which would be needed for evidence-based facility specification. Seventh, regulatory, workforce, and cost-effectiveness aspects of CEU implementation are beyond the scope of this analysis and require dedicated prospective evaluation, including discrete-event simulation of facility throughput. Eighth, although this analysis was framed as multicenter, the de-identified extraction did not preserve hospital-level identifiers, precluding a formal center-stratified comparison; future work using identified extractions should examine inter-site variability explicitly.

## 5. Conclusions

This multicenter retrospective feasibility assessment found that approximately 28.6% of ED visits involved patients with low-acuity final KTAS classifications potentially suitable for management within a Compact Emergency Unit framework. These patients were characterized by a markedly shorter median length of stay (92 vs. 171 min; Cliff’s δ = −0.51, large effect), a near-universal symptomatic home discharge rate (98.8%), and a low specialty consultation burden (median 0 per visit). The hour-by-hour temporal analysis showed a 20:00 evening peak and substantial sustained nighttime demand (43.7% of CEU-eligible visits), providing concrete epidemiological support for a 24 h operational model.

Machine-learning analysis achieved moderate-to-good discrimination (best AUROC 0.794 for vital-sign-augmented random forest) with near-perfect XGBoost calibration (ECE 0.020). A combined ML-or-abnormal-vital-sign screening rule raised non-discharge sensitivity to 94.1%, and the sensitivity analysis revealed that the standard in-ED re-triage workflow already catches 40% of non-discharge events upstream, together supporting a layered safety architecture that combines parsimonious modeling, structured vital-sign screening, and preserved re-triage capability.

This work should be regarded as a preliminary feasibility assessment establishing the epidemiological prerequisites for the CEU concept. Whether such facilities can safely and effectively absorb this patient volume remains a question that can only be answered by prospective implementation studies with structured outcome follow-up, simulation-based capacity planning, and formal regulatory and workforce evaluation.

## Figures and Tables

**Table 1:** Demographic and operational characteristics of CEU-eligible and non-eligible patients (final triage classification, *n* = 34,544). Effect sizes: Cliff’s delta for continuous variables (small ≥ 0.147, medium ≥ 0.33, large ≥ 0.474); Cramér’s V for categorical variables.

Variable	CEU-Eligible (*n* = 9871)	Non-Eligible (*n* = 24,673)	*p*-Value	Effect Size
Age (years), median (IQR)	56 (37–71)	63 (46–76)	<0.001 †	Cliff’s δ = −0.16 (small)
Female sex, *n* (%)	5635 (57.1)	13,476 (54.6)	<0.001 ‡	V = 0.022 (negligible)
Medical aid insurance, *n* (%)	570 (5.77)	1419 (5.75)	0.954 ‡	V = 0.0003 (negligible)
Length of stay, min, median (IQR)	92 (45–146)	171 (113–280)	<0.001 †	Cliff’s δ = −0.51 (large)
Specialty consultations, median (IQR)	0 (0–1)	1 (0–1)	<0.001 †	Cliff’s δ = −0.25 (small)
Symptomatic home discharge, *n* (%)	9753 (98.8)	22,366 (90.6)	<0.001 ‡	V = 0.14 (small)
Nighttime visit (20:00–07:59), *n* (%)	4316 (43.7)	8510 (34.5)	<0.001 ‡	V = 0.086 (negligible)
Severe emergency disease (MOHW), *n* (%)	353 (3.58)	4374 (17.7)	<0.001 ‡	V = 0.18 (small)

† Mann–Whitney U test; ‡ Chi-squared test. IQR = interquartile range; MOHW = Korean Ministry of Health and Welfare severe emergency disease classification. Effect size interpretation for Cramér’s V: negligible < 0.1, small < 0.3, medium < 0.5, large ≥ 0.5.

**Table 2:** Machine-learning model performance for non-discharge prediction among CEU-eligible patients (*n* = 9871; 118 events; patient-level 5-fold cross-validation with StratifiedGroupKFold). Results shown for both the original 16-feature configuration and the vital-sign-augmented 22-feature configuration. AUROC = area under the receiver operating characteristic curve; AUPRC = area under the precision–recall curve for the non-discharge class; ECE = expected calibration error (10 equal-width bins). Sensitivity and specificity reported at the Youden-optimal threshold. Prevalence-only Brier baseline = 0.0119.

Model	Feature Set	AUROC	95% CI	Sensitivity	Specificity	AUPRC (Non-dc)	Brier	ECE
Logistic Regression	16-feature	0.713	0.670–0.757	80.5%	52.4%	0.030	0.157	-
Random Forest	16-feature	0.668	0.618–0.712	61.0%	72.0%	0.037	0.073	-
XGBoost	16-feature	0.675	0.628–0.721	85.6%	42.0%	0.022	0.073	-
Logistic Regression	+vitals (22)	0.740	0.702–0.781	74.6%	62.6%	0.062	0.148	0.222
Random Forest	+vitals (22)	0.794	0.758–0.829	83.1%	67.2%	0.061	0.024	0.053
XGBoost	+vitals (22)	0.717	0.672–0.760	81.4%	55.9%	0.052	0.018	0.020

Sensitivity/specificity at Youden-optimal threshold. DeLong pairwise: LR vs. RF: z = −3.565, *p* < 0.001; LR vs. XGBoost: z = −4.661, *p* < 0.001; RF vs. XGBoost: z = −0.924, *p* = 0.356. Prevalence-only Brier baseline = 0.0119. Note: high PPV values (~99%) reflect class prevalence (home discharge = 98.8%) rather than model discrimination.

## Data Availability

The datasets used in this study are not publicly available due to institutional data governance requirements but are available from the corresponding author upon reasonable request and subject to institutional approval.

## Abbreviations

AUROC	Area under the receiver operating characteristic curve
CEU	Compact emergency unit
CI	Confidence interval
CAN	Certified nursing assistant
ED	Emergency department
EMT	Emergency medical technician
IQR	Interquartile range
KTAS	Korean Triage and Acuity Scale
LOS	Length of stay
NPV	Negative predictive value
PPV	Positive predictive value
RN	Registered nurse
SHAP	Shapley additive explanations
SMOTE	Synthetic minority oversampling technique
